# Pathophysiological roles of integrins in gliomas from the perspective of glioma stem cells

**DOI:** 10.3389/fcell.2022.962481

**Published:** 2022-09-16

**Authors:** Maoyu Wang, Sen Shen, Feng Hou, Yaohua Yan

**Affiliations:** Department of Neurosurgery, Affiliated Hospital of Nantong University, Medical School of Nantong University, Nantong, China

**Keywords:** integrin, glioma, glioblastoma, glioma stem cell, targeted therapy

## Abstract

Glioblastoma is the most common primary intracranial tumor and is also one of the most malignant central nervous system tumors. Its characteristics, such as high malignancy, abundant tumor vasculature, drug resistance, and recurrence-prone nature, cause great suffering to glioma patients. Furthermore, glioma stem cells are the primordial cells of the glioma and play a central role in the development of glioma. Integrins—heterodimers composed of noncovalently bound *a* and *ß* subunits—are highly expressed in glioma stem cells and play an essential role in the self-renewal, differentiation, high drug resistance, and chemo-radiotherapy resistance of glioma stem cells through cell adhesion and signaling. However, there are various types of integrins, and their mechanisms of function on glioma stem cells are complex. Therefore, this article reviews the feasibility of treating gliomas by targeting integrins on glioma stem cells.

## Introduction

### Glioblastoma

Glioblastoma (GBM) is the most common primary malignant brain tumor, representing approximately 57% of all gliomas and 48% of all primary malignant central nervous system (CNS) tumors (Tan et al., 2020). Glioblastoma multiforme is the most common and aggressive primary malignant CNS tumor in adults (Huang et al., 2020). Aggressive tumor growth correlates with a short median overall survival (OS) that oscillates between 14 and 17 months (Litak et al., 2019). The current treatment modalities for GBM are mainly maximum safe tumor resection, postoperative radiotherapy, and chemotherapy (Li et al., 2020; Weller and Le Rhun, 2020). Unfortunately, despite more than two centuries of technological advances in the treatment of glioma, the death rate associated with GBM patients remains exceptionally high, especially due to high GBM recurrence and drug resistance.

### Glioma stem cells (GSCs)

In recent years, cancer stem cells (CSCs) have come to the forefront and have become a target for the treatment of malignant tumors. CSCs are a subpopulation of tumor cells with stem cell properties characterized by their self-renewal ability and tumor proliferation potential (Biserova et al., 2021) and their possession of embryonic or tissue stem cell genes (Chen et al., 2012; Shibue and Weinberg, 2017).

Many studies have shown that a small proportion of cells in gliomas have been identified to be having functional and phenotypic similarities to neural stem cells; these are known as GSCs (Galli et al., 2004; Singh et al., 2004; Tirosh et al., 2016) or glioma-initiating cells (GICs) (Tabatabai and Weller, 2011; Lathia et al., 2015; Yi et al., 2019; Biserova et al., 2021). Neural cancer stem cells and central nervous system tumor stem cells, including GSCs, can maintain their unique cellular stemness and various malignancies and are also associated with the tumor microenvironment or niche (Lathia et al., 2011). The microenvironment includes the vasculature, various infiltrating and resident immune cells, other glial cell types, and glioma cells in addition to GSCs. These microenvironmental niches also exhibit various forms of signaling, such as direct contact or paracrine signaling. These signals ensure that tumor cell and GSC growth is not monitored and that these cells are not destroyed by the immune system (Hambardzumyan and Bergers, 2015). In addition to their functions, such as self-renewal, ability to differentiate into multiple cell lineages, proliferation potential, and tumor angiogenesis, GSCs exhibit strong therapeutic resistance (Brooks and Parrinello, 2017; Ruiz-Garcia et al., 2020; Suva and Tirosh, 2020; Biserova et al., 2021) and can resist conventional chemotherapy and radiation therapy through DNA repair (Bao et al., 2006a; Huang et al., 2010).

In summary, glioma stem cells are likely to rely on these abilities to survive after treatment and eventually lead to tumor recurrence. Biserova et al. (2021) noted an association between glioma stem cells and the development of glioma recurrence. In addition, GSCs are at the apex of an entropic hierarchy (Prager et al., 2020) and are also considered to be the basis of gliomagenesis (Nakada et al., 2013). Some researchers have proposed using the cell expression molecule CD133 as a screening tool. This is because the glioma subpopulation of CD133 shows a greater ability to self-renew, proliferate, and form tumors *in vitro* while retaining the homogenous histological characteristics of the original donor (Singh et al., 2004). Interestingly, however, some CD133^-^ glioma cells have been reported to have a malignant phenotype with stronger tumor-promoting potential (Beier et al., 2007). In addition, GSCs showed CD15, CD36, CD44, and CD49f/integrin α6 markers, which were also expressed on normal neural stem cells (NSCs) (Ma et al., 2018).

### Integrins

Integrins are protein complexes that link the extracellular matrix (ECM) to the actin-based cytoskeleton and were first proposed by Tamkun et al. (1986) in 1986. Integrins are present in many organisms and are critical molecules involved in cell‒cell and cell–microenvironment communication (Janiszewska et al., 2020). Integrins are heterodimerized in the endoplasmic reticulum (Mchugh et al., 2010; Dransart et al., 2022). They can constitute the principal adhesion receptors for the extracellular matrix (ECM) (De Franceschi et al., 2015). The binding of unique *a* and *ß* subunits determines the functional specificity of the receptor (Takada et al., 2007). Integrins can be classified into four categories: LDV-binding integrins, which bind to an acidic amino acid motif (referred to as ‘LDV’); A-domain β1 integrins; non αA-domain-containing laminin-binding integrins; and RGD (Arg-Gly-Asp)-binding integrins (Humphries et al., 2006; Anderson et al., 2014b). Integrins possess different conformational states, a feature that determines the affinity of integrins for ligands. A bent (closed) integrin represents the inactive form and has a low affinity for ECM ligands. In contrast, a fully extended (open) integrin is active and can trigger downstream signaling and cellular responses upon ligand binding (Markovic-Housley and Garavito, 1986). Integrins represent a complex and highly dynamic mechanism responsible for regulating various aspects of cell fate, such as survival, migration, polarity, and differentiation (Shen et al., 2012; Anderson et al., 2014a). Thus, integrin-mediated adhesion and signaling are precursors to the pathogenesis of many human diseases, including bleeding disorders, cardiovascular disease, and cancer (Winograd-Katz et al., 2014).

**FIGURE 1:**
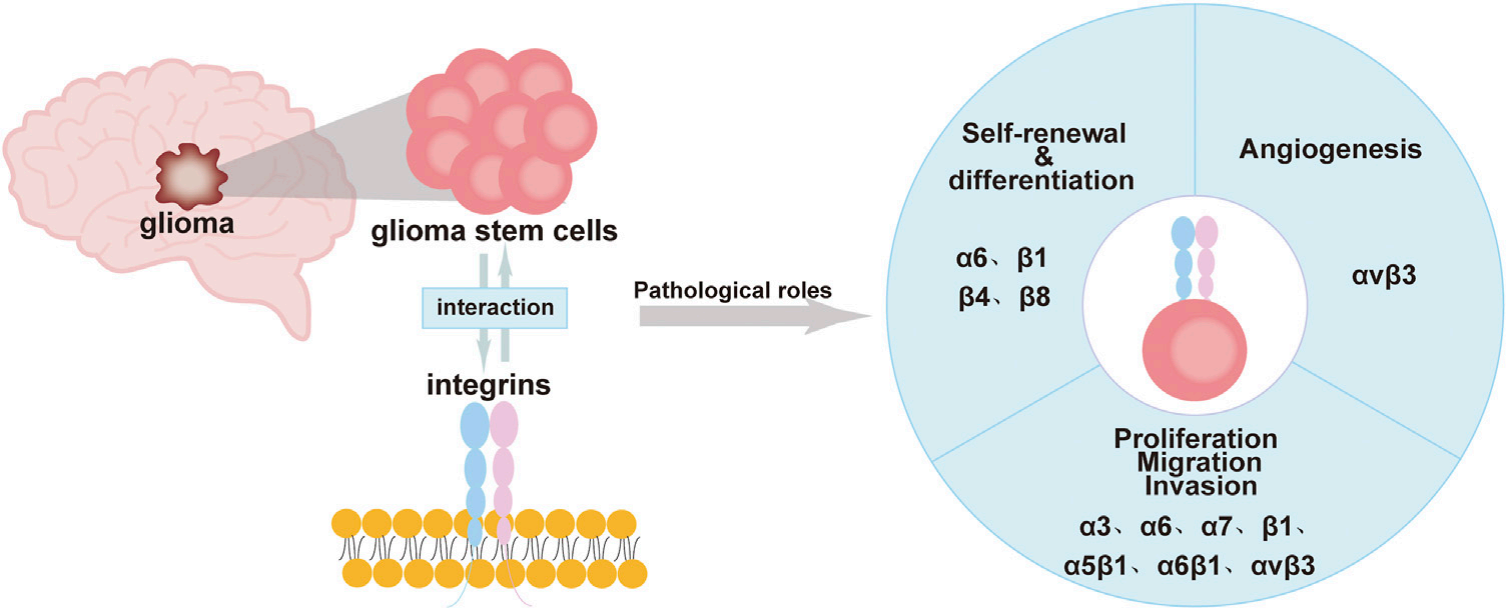
Roles of integrins in GSCs including self-renewal, differentiation, immune regulation, proliferation, migration, invasion, and angiogenesis.

Integrins are expressed at high levels in GSCs and have a “bridging” role. Most integrins transmit intracellular/extracellular cell signaling/communication and are involved in maintaining the stemness characteristics and functions of GSCs. This includes the self-renewal and differentiation of GSCs, invasion, migration, and the tumor microenvironment of gliomas (Bello et al., 2001; Nakada et al., 2013; Herrmann et al., 2020; Tao et al., 2020). Currently, drugs that inhibit integrins, such as cilengitide, have been found to treat gliomas by inhibiting the activity of GSCs or increasing the effect of autophagy (Lomonaco et al., 2011; Yu et al., 2018). This review will provide a more systematic account of the effects of integrins on GSCs. We hope it provides new ideas and directions for glioma-targeted therapy (Figure 1; Table 1).

**TABLE 1:** Pathophysiological role of integrins in GSCs in glioma.

Integrin	Category	Self-renewal and differentiation	Angiogenesis	Proliferation, migration, and invasion	Reference
α3	Laminin-binding integrins	NO	NO	YES	(D’abaco and Kaye, 2007; Nakada et al., 2013; Wu et al., 2021)
α6	Laminin-binding integrins	YES	NO	YES	(Velpula et al., 2012; Hale et al., 2014; Ying et al., 2014; Ma et al., 2016; Tilghman et al., 2016; Herrmann et al., 2020; Tao et al., 2020)
α7	Laminin-binding integrins	NO	NO	YES	Haas et al. (2017)
β1	A-domain β1 integrins	YES	NO	YES	(Edwards et al., 2011; Manini et al., 2019; Saleh et al., 2019; Tao et al., 2020; Seguin et al., 2021)
β4	RGD-binding integrins	YES	NO	NO	Ma et al. (2019)
β8	RGD-binding integrins	YES	NO	NO	Guerrero et al. (2017)
αvβ3	RGD-binding integrins	NO	YES	YES	(Friedlander et al., 1995; Bello et al., 2001; Naik et al., 2003; Bao et al., 2006b; Cheresh and Stupack, 2008; Schnell et al., 2008; Peddibhotla et al., 2013; Roth et al., 2013; Ruiz-Ontanon et al., 2013; Mikheev et al., 2015; Burgett et al., 2016; Wick et al., 2016)
α5β1	A-domain β1 integrins	NO	NO	YES	Siney et al. (2017)
α6β1	A-domain β1 integrins	NO	NO	YES	Tao et al. (2020)

## Integrins promote glioma progression by acting on GSCs

### Integrins are involved in the self-renewal and differentiation of GSCs

It is well known that GSCs are characterized by their tumorigenic entity and self-renewal, as well as differentiation ability (Nakada et al., 2013). Yu et al. (2018) demonstrated that integrins binding to fibronectin (FN) can be increased in a concentration-dependent manner induced by matrix metallopeptidase (MMP)-2 and MMP-9, which in turn activate the FAK/paxillin/AKT signaling pathway, leading to decreased levels of GSC markers such as SOX2 and Nestin, along with increased levels of glial fibrillary acidic protein (GFAP) and *ß*-tubulin. SOX2 plays an important role in the maintenance and self-renewal capacity of GSCs (Chen et al., 2021). In addition, GFAP and *ß*-tubulin are differentiation-related markers (Perez et al., 1988; Wang et al., 2020b).

Among integrins, α6 is a key molecule for GSC self-renewal and differentiation and is also a GSC marker and invasion promoter. Integrin α6 is widely expressed in four malignant cell states in GBM (neural progenitor cell (NPC)-like, oligodendrocyte progenitor cell (OPC)-like, astrocyte (AC)-like, and mesenchymal stromal cell (MES)-like states). In addition, glioma cells with higher integrin α6 expression are able to form tumors in a shorter period of time (Lathia et al., 2010; Tao et al., 2020). A study by Hale et al. (2014) proposed that integrin α6 on GSCs is coexpressed with the malignancy marker CD36, with the former decreasing with the latter and progressive loss of its self-renewal and tumorigenic capacity (Hale et al., 2014). Similarly, integrin α6 inhibition by Kruppel-like factor 9 (KLF9) reduced stemness and laminin-dependent GBM neurosphere cell adhesion and cell migration in GBM. This implies that the inhibition of integrin α6 may have antitumor effects (Ying et al., 2014; Ma et al., 2019). Interestingly, the upregulation of laminin-binding integrin α6 in the 3D environment not only increases the expression of GSC markers but also promotes the activation of stemness signaling pathways (Ma et al., 2016). Furthermore, α6β1, formed by the binding of two subunits of integrin α6 and β1, acts as a signaling receptor for WISP1 to participate in the autocrine loop of GSC proliferation and self-renewal (Tao et al., 2020).

Integrins β4 and β8 also act in the self-renewal action of GSCs. Ma et al. found that integrin β4 expression is increased in GSCs and glioma tissues by mRNA sequencing analysis. In addition, integrin β4 also correlates with glioma grading, as determined by *in vitro* spheroid assays. When integrin β4 was knocked down, the number and sphere-forming rate of CD133^+^ GSCs were significantly reduced (Ma et al., 2019). Similarly, when GSCs contain low levels of integrin β8, not only is the sphericity rate low but also markers of GSCs, such as CD133 and SOX2, are reduced (Guerrero et al., 2017).

Notably, Barnes et al. also found that the integrin β1-linked glycocalyx protein signaling pathway induces a mesenchymal stem cell phenotype in GBM. Inhibiting integrin-ECM signaling or glycoprotein bulkiness ultimately acts as a therapeutic inhibitor of GBM (Barnes et al., 2018). All of the aforementioned findings suggest that integrins in glioma stem cells directly or indirectly contribute to the self-renewal and differentiation capacity of GSCs.

### Translation of integrins affects GSC proliferation, migration, and invasion

In addition to accelerating the self-renewal and differentiation process of GSCs, integrins are also involved in the development of gliomas, which is reflected by promoting the proliferation, migration, and invasion of GSCs.

In these studies, the upregulation of integrin α3 expression was associated with GSC invasion. The researchers found that integrin α3 was not only localized in GBM but was also found around invading cells and blood vessels. This is due to integrin α3 mediating the ERK1/2 signaling pathway, which enhances GSC invasion (Nakada et al., 2013). Interestingly, Wu et al. showed by survival analysis of GSCs that integrin α3 was associated with a significantly longer survival time in GBM patients. The data suggest that low levels of integrin α3 expression are positively associated with prolonged survival (Wu et al., 2021). In addition, integrin α7 can also act on proliferation. Haas et al. suggested that the expression of integrin α7 in normal human neural progenitor cells (NHNPs) was significantly lower than that in GSCs. Inhibition of integrin α7 affects the proliferation of GSCs. This is because by silencing the gene for integrin α7, laminin-induced activation of signaling proteins such as FAK, AKT, and Src can be inhibited (Haas et al., 2017). Moreover, integrin α6 plays an important role in tumor invasion, survival, malignancy, and drug resistance. In 2012, Velpula et al. (2012) showed that the interaction of integrin α6 and N-calcineurin could modulate the invasive effects of GSCs through the ERK signaling pathway. Herrmann et al. also showed that in high-grade glioma cells, the integrin α6-FAK signaling pathway increased the downstream signal transducer and activator of transcription 3 (STAT3), transcription factor 13 (TET3), and 5-hydroxymethylcytosine (5 hm C) activities and expressions. Upregulation of this pathway also leads to hydroxy methylation of genes that are important for GSCs, ultimately resulting in maintaining high survival and proliferation rates of GSCs and promoting malignant phenotypes and drug resistance in GSCs (Herrmann et al., 2020). Inhibition of integrin α6 can affect ERK, FAK, and other signaling pathways, thereby reducing the high drug resistance and malignant phenotypic transformation of GSCs (Tilghman et al., 2016).

In addition, integrin β1 and integrin β8 also play an important role in the movement and growth of GSCs. Seguin et al. found that integrin β1 co-localizes with galectin-3 (Gal-3) in GSCs, and their experiment demonstrated that knocking down integrin β1 significantly inhibits macropinocytosis effects. Gal-3/RAB10 (a member of the Ras superfamily of small GTPases)/integrin β1 promotes PI3K/Akt downstream signaling to stimulate macropinocytosis and reveals that integrin β1 provides favorable conditions for GSCs’ survival, invasion, and tumorigenic ability (Seguin et al., 2021). Similarly, the 3D nanofiber scaffold developed by Saleh et al. protects against GSCs’ invasion by regulating integrin β1 and Gal-3 expression (Saleh et al., 2019). In addition, Edwards suggested that activation of the connective tissue growth factor (CTGF)-integrin β1-TrkA complex formed in GSCs could increase the invasiveness of GBM (Edwards et al., 2011). Moreover, Manini et al. showed in an *in vitro* model that integrin β1 on the surface of GSCs binds directly to a ligand called premigratory protein-SEMA7A. Integrin acts as an intermediate receptor to trigger FAK signaling and phosphorylate it, thereby promoting cytoskeletal reorganization and cell motility in GSCs (Manini et al., 2019). Interestingly, data from Vehlow et al. (2017) showed that dual inhibition of β1 integrin and JNK was effective in enhancing GSC eradication when treated with concurrent radiotherapy and chemotherapy. Malric et al. demonstrated that integrin β8 could also be a marker of glioma grade, is highly expressed in GSCs, and positively correlates with SOX2. Silencing integrin β8 reduced the sphere-forming and migratory abilities of GSCs and cell adhesion. Integrin β8 can maintain GSC growth by reducing apoptosis so that integrin β8 inhibition can induce a significant increase in caspase-dependent GSC apoptosis and increase the efficacy of radiotherapy (Malric et al., 2019).

Integrin αvβ3, as one of the widely studied integrins, has been shown to be involved in the migration and proliferation of GSCs. Ruiz-Ontanon et al. (2013) revealed that integrin αvβ3 and low levels of cytoplasmic p27 and its downstream effector proteins Rac and RhoA GTPases provide GSCs isolated from tumor peripheral regions with more migratory capacity and infiltration into adjacent tissues. Moreover, tumor-associated macrophages (TAMs) and GSCs are located in the perivascular region in large numbers (Ye et al., 2012; Pietras et al., 2014). Interestingly, the interaction between GSCs and TAMs was involved in the regulation of GSC proliferation. This interaction is due to the binding of the periosteal protein (POSTN) secreted by GSCs to integrin αvβ3 of TAMs (Zhou et al., 2015). Integrin αvβ3 on TAMs acts as a receptor for POSTN. Mikheev et al. also showed that the binding of integrin αvβ3 to POSTN can cause adhesion and migration of GSCs and can promote the growth of GSCs by activating the FAK signaling pathway. The binding of integrin αvβ3 to POSTN can also inhibit the cytotoxic effect of cilengitide (an inhibitor that can inhibit integrin αvβ3) on GSCs (Mikheev et al., 2015). Similarly, cilengitide can reverse the effect on FN that can modulate GSCs in terms of cell adhesion, proliferation, and differentiation, making GSCs more chemoresistant to alkylating agents. Thus, they demonstrated the involvement of integrin αvβ3 in the regulation of GSCs by the AKT pathway (Yu et al., 2018). These results suggest that integrin αvβ3 can be involved in the migration and proliferation of GSCs. In addition, integrin α5β1 interacts with recombinant A disintegrin and metalloprotease 10 (ADAM10) or recombinant A disintegrin and metalloprotease 17 (ADAM17) and adheres to FN, exerting an adhesive role in GSCs and promoting GSC migration through this adhesion (Siney et al., 2017), while integrin α6β1 binds to WISP1 secreted by GSCs and promotes GSC proliferation through Akt-activated phosphorylation (Tao et al., 2020).

### Integrins contribute to tumor angiogenesis in GSCs

GBM is a highly malignant brain tumor with an extensive and abnormal tumor vasculature, including multiple types of blood vessels (Shao et al., 2015). A high angiogenic phenotype is a prominent feature of GBM and is thought to contribute to the aggressive growth and tumor recurrence of these tumors (Ahluwalia and Gladson, 2010; Onishi et al., 2011; Shao et al., 2015). Bao et al. (2006b) suggested that GSCs are more likely to form in the tumor vasculature than non-tumor stem cell gliomas under the same conditions. In addition, GSCs can also promote tumor angiogenesis through VEGF and stromal-derived factor 1 (SDF-1) (Folkins et al., 2009). However, multiple integrins are also involved in the angiogenesis of GBM.

Integrin αvβ3 is alleged to be involved in angiogenesis in GSCs, and integrin αvβ3 and integrin αvβ5 are key regulatory molecules of the tumor microenvironment that are highly expressed not only in gliomas but also in glioma vessels (Bello et al., 2001; Schnell et al., 2008; Roth et al., 2013). In turn, the tumor microenvironment can elevate the expression of the vascular endothelial growth factor and promote the formation of blood vessels from endothelial precursors in GSCs (Bao et al., 2006b). The direct intercellular contact that occurs through the binding of αvβ3 on vascular endothelial cells to RGD peptides in the extracellular structural domain of L1 cell adhesion molecules (L1CAM) on GSCs in the presence of bFGF triggers the activation of bone marrow tyrosine kinase on chromosome X (BMX), FAK, and P130 Crk-associated substrate (p130CAS) on bone marrow X chromosome, resulting in migration (Peddibhotla et al., 2013; Burgett et al., 2016). Integrin αvβ3 has been reported to bind to the basic fibroblast growth factor (bFGF), thus promoting angiogenesis (Friedlander et al., 1995; Naik et al., 2003; Cheresh and Stupack, 2008).

## Combination therapies with integrins

Currently, targeted integrins have not achieved significant efficacy in the treatment of gliomas at the clinical stage. However, we still believe that targeted integrins are feasible for treating gliomas by killing or reducing GSC proliferation, differentiation, self-renewal, and drug resistance.

### Virus targets integrins on GSCs for therapeutic effect

Zika virus (ZIKV) could have a therapeutic effect on integrin avβ5, which is highly expressed in GBM tissues and expressed at low levels in other normal tissues (Bello et al., 2001; Zhao et al., 2016), and can be used as a stemness marker for glioma (Wang et al., 2020a). Interestingly, ZIKV, a mosquito-borne positive-stranded RNA virus of the family Flaviviridae (genus *Flavivirus*) (Song et al., 2017), can preferentially target neural precursor cells for infection (Zhu et al., 2020). Zhu et al. concluded that the SOX2-integrin avβ5 axis can promote the killing of GSCs by ZIKV. Silencing integrin avβ5 reduces the infection effect of ZIKV (Zhu et al., 2020).

In addition, Berghauser Pont’s team pointed out that the adenovirus Delta24-RGD can enter cells *via* integrin αvβ3/αvβ5. However, glioblastoma has a different sensitivity to Delta24-RGD. In contrast, novel histone deacetylase inhibitors (HDACis), such as LBH589 (panobinostat) and SCRIPTAID, affect integrin αvβ3/αvβ5 and share a common cell death pathway with Delta24-RGD. Overall, Delta24-RGD can enhance the antitumor capacity in GSCs (Balvers et al., 2014; Berghauser Pont et al., 2015). In addition, Przystal et al. proposed that the recombinant adeno-associated virus genome (rAAV) binds to a phage to form an adeno-associated virus and phage (AAVP). Then, integrin αvβ3 can bind to the double-cyclic CDCRGDCFC (RGD4C) ligand and internalize RGD4C/AAVP (Tsafa et al., 2020). Of course, the αvβ5 heterodimer can also bind RGD4C but to a lower extent than αvβ3. After RGD4C and integrin binding, they can be therapeutically effective *in vitro* by targeting GSC gene delivery and expression (Przystal et al., 2019).

### Inhibitors of integrins—Synthetic peptides

Cilengitide, a “cyclic-RGD segmental peptide,” can inhibit integrins αvβ3 and αvβ5 and prevent them from binding to ECM proteins such as vitronectin (VN) and FN (Burke et al., 2002; Albert et al., 2006). Therefore, cilengitide can inhibit the adhesion of integrins to the ECM and ultimately inhibit glioma proliferation, migration, and angiogenesis. Antitumor effects against gliomas were demonstrated in relevant clinical studies (Tabatabai et al., 2010). Interestingly, cilengitide is also involved in GSC inhibition. Lomonaco et al. showed experimentally that cilengitide could inhibit GSC self-renewal by inducing autophagy and thus reducing tumor cell survival. They also indicated that cilengitide might also sensitize GSCs to *γ*-radiation. This was supported by the presence of green fluorescent protein (GFP)-LC3 (a signature protein on autophagosomal membranes) spots and increased expression of LC3II and increased autophagic vacuole (AV) formation (Lomonaco et al., 2011). In addition, as mentioned previously, integrins can interact with FN in the ECM in terms of adhesion to GSCs. It has also been specified that cilengitide can also inhibit the reaction of integrins in GSCs with FN and inhibit the expression of p-ERK1/2 and cyclin D1 *via* the FAK/paxillin/AKT signaling pathway. Thus, cilengitide can inhibit the biological behavior of GSCs in terms of cell adhesion, proliferation, and differentiation. The article also indicates that cilengitide can reverse FN adhesion, leading to chemoresistance to carmustine (Yu et al., 2018). Furthermore, Flavahan et al. showed that Glut-3 (glucose transporter 3) addiction is also a feature of GSCs. They hypothesized that cilengitide could target this feature and achieve eradication of the most aggressive and drug-resistant GSCs (Flavahan et al., 2013). In addition, Dahmani et al. also reported that integrin αv on GSCs binds to CD9 and CD103 on NK cells, resulting in NK-cell dysfunction and ultimately inhibiting the killing of GSCs by NK cells. However, cilengitide significantly enhanced the antitumor activity of NK cells *in vivo* by inhibiting integrin αV (Dahmani and Delisle, 2018).

In *in vitro* and *in vivo* animal models, small-molecule integrin antagonists (SMIAs) were identified to modulate migration and apoptotic processes in glioma cell lines (Russo et al., 2013). However, Paolillo et al. pointed out that a small-molecule integrin-rgd antagonist (SMIA 1a-RGD) could act on RGD-binding integrins, which recognize the RGD sequence present in components of the extracellular matrix. These integrins serve a crucial function in the dissemination of GSCs and are overexpressed in GBM. In addition, the viability of GSCs treated with 25 μm SMIA 1a-RGD for 48 h was significantly reduced, accompanied by a decrease in FAK and AKT expressions. Interestingly, Paolillo et al. speculated that this phenomenon may be related to the inhibition of GSC migration and cysteine-dependent loss-of-nest apoptosis by SMIA 1a-RGD (Paolillo et al., 2018).

### CAR-T cells with integrins

The integrin αvβ3 axis plays a key role in POSTN-mediated TAM recruitment (Zhou et al., 2015). Interestingly, a recent study by Cobb et al. pointed out that this immunotherapy by CAR-T cells targeting integrin αvβ3 and POSTN complexes can inhibit the effective treatment of glioma cells. CAR-T cells targeting integrin αvβ3 are highly efficient *in vivo* and can reduce glioma growth (Cobb et al., 2022). Thus, the site where GSC-secreted POSTN binds to integrins may be a potential target for the design of effective immunotherapies to improve the survival of GBM patients (Shi et al., 2015; Zhou et al., 2015). However, it could be stated that one caveat to treatments such as CAR-T cells is that the reason that gliomas recur is the ability of GSCs to invade normal surrounding brain tissue and reside behind the blood–brain barrier and, thus, escape the immune system that operates in most of the body (Kubo and Takakura, 2002). This would make CAR-T therapy less effective or ineffective against GBM recurrence.

## Conclusion

Integrins are widely expressed in most GSCs as “bridge” proteins. Integrin-mediated signaling pathways can lead to adhesion and self-renewal, differentiation, motility, and angiogenesis, which are characteristics of tumor stem cells. In conclusion, most integrins maintain the survival and stemness characteristics of GSCs. Therefore, we can use various properties of integrins to target therapies precisely. However, the variety of integrins and their complex mechanisms manifest different roles in different pathways. Most integrins are upregulated, leading to continued GSCs’ growth, motility, and maintenance of the stem phenotype. However, some integrins can promote the entry of adenoviral complexes into GSCs and produce killing effects. How can the targeting of gliomas be achieved by inhibiting integrins and promoting integrins as drug ligands? We need to further increase our understanding of the oncogenic mechanism of integrins in gliomas to classify the effects of integrins and apply the binding sites of these integrins to design targeted drugs. However, some integrins have been targeted as therapeutic targets for gliomas. In future work, further understanding of the oncogenic mechanisms of integrins in glioma needs to be developed. Second, the binding sites of these integrins can also be applied to design targeted drugs, thus increasing the degree of killing of GSCs and ultimately improving the treatment of GBM. Therefore, we believe that using integrin targeting of GSCs in the treatment of gliomas is a method worthy of further research.
